# A Large Tumour of the Mamma, Spontaneously Cured

**Published:** 1847-02

**Authors:** A. B. Greene

**Affiliations:** Sumter County, Georgia


*A large Tumour of the Mamma, spontaneously cured.* Reported by A. B. Greene, M. D., of Sumter County, Georgia.—The subject of these remarks is a negro woman belonging to Col. M. A short time after she arrived at the usual age for the full development of the sexual organs, the right mamma was observed to be largest, and increased in dimensions gradually and continuously, unattended with pain, except what was induced by its suspension, when not properly supported. About twelve months preceding her pregnancy, my attention was first called to an examination of the enlarged mamma, and I found all the usual evidences of simple sarcoma present, with nothing of a malignant character. The skin and subjacent tissues seemed as healthful to the touch, and as free from uneasiness when handled, as any other mass of flesh about the body. Its size already amounted to great deformity, and was obvious to any passing observer.

I did not hesitate to advise her intelligent owner, that an immediate operation was the only certain cure; and pointed out the danger should amputation be deferred, that might result from pregnancy and accouchement, in the development of some malignant tendency, or at least, subjection to severe suffering.

The force of my suggestions was appreciated; but fearing the operation, and trusting to some happy, fortuitous change, it was neglected. She became pregnant, and at the full term of gestation in January last was delivered without any unusual difficulty, and soon felt pain in the enlarged breast for the first time. At the expiration of six weeks, it had grown so rapidly, that while sitting upright the enlarged mamma rested on her lap. It was enormously distended, and of such weight, that however well supported, her chest was strongly inclined forward. I could never detect any circumscribed induration, nor any other evidence of the formation of abscess. She was put upon strict antiphlogistic treatment, medicinal and dietetic. General and local depletives, emollient fomentations and poultices, to relieve turgidity; and the occasional application of the pump and cup to the nipple to encourage a discharge of the milk, should any possibly be secreted.

While these means were being applied, a spontaneous discharge occurred from about an inch above the nipple—in a continued stream it incessantly poured out, until one gallon and a half of a milk-like fluid had come away. Immediate and complete relief was expressed, and no appearance of debility, or other adverse symptom, as a consequence of the sudden and almost incredible quantity dislodged, was observed.

From that time, the breast gradually assumed the size it had attained before her accouchement. Her general health and strength improved daily, and in a short time she was out on the farm at labor, though of an easy kind, and self-imposed. While thus employed, she was exposed to a drenching rain, and in a few days I was summond to visit her again.

I found her now labouring under an acute rheumatism, in its most dan ger os and painful form. The pleura was implicated, and a manifest tendency to low typhus rendered her condition very critical.

When we found that she might possibly survive the attack, my attention was again directed to the condition of the diseased breast. Upon examination an ulcer was found, situated near the nipple—the breast again greatly increased in size. The ulcer discharged matter of a thin purulent character, and most noisome odour. Other ulcers were formed, and they soon numbered seven, emitting vast quantities of insupportable horrid pus-like fluid. By patient perseverance in the use of the chloride of lime, charcoal poultices, and a variety of astringent injections, the discharges assumed a more healthy appearance and less offensive odour.

The patient was now a miserable looking object of emaciation ; and to every one her death seemed inevitable. It would be needless to dwell upon the treatment here, constitutional and local.

The largest and most pendent ulcer presented a prominence in the centre, which I found to be insensible. Upon raising it with the forceps, I was surprised to find that by gentle traction a considerable mass was drawn out without any pain. I applied the scissors and separated that portion. There was not a drop of blood discharged. I then seized the remaining mass with a small tenaculum, and readily drew out in a string-like form, a connected volume of flesh coloured substance, until by the pain produced, I supposed the mass to be too large to pass through the ulcer, and again detached it with the scissors.

I now found a large hollow chasm left under the ulcer, and changed the position of the breast to examine the other ulcerated openings, but as they were situated above, nothing unusual presented at them ; but when restored to the same situation again, I found the chasm occupied, and another mass presenting. By pulling gently with the forceps at different points, I succeeded in starting another slough through the ulcer, which passed easily, until one entire mass of large size fell from it without the use of any excising instrument. No blood followed, except what escaped from the edges of the dilated ulcer.

There was now an extensive, unoccupied space, left by the large volume of detached slough ; and by the aid of the light admitted through the several ulcerated openings, (I fancied,) was seen the original "bona fide" gland, bounding the upper part of this space.

As nature had performed the amputation so well, thus far, I determined not to interfere, in the already exhausted condition of the patient, by removing the now redundant skin.

Our next care was to ascertain the amount of the self-amputated mass, which was attached to the hook of a pair of "draw-spring steelyards, (the only means of weighing at hand) and weighed one and a half pounds. The small fragments, not weighed, Col. M. supposed would amount to at least a half pound more.

We were surprised, at first, that so much bulk and volume should weigh no more ; but upon careful examination, I found it composed of a cellular structure, comparable to honey-comb, interspersed with fine particles of a "coffee-ground" colour. The breast was bathed with infusion of red-oak bark, and covered with a large poultice of the same excellent astringent, moistened with solution chloride of lime, and secured by means of bandages, bound on so as to exert due compression on the now flaccid parts. General directions were given for future management, and I did not see the patient from that time, which was 24th of April last, until a few days since, when I visited her, in order to report upon her present condition.

She has been doing the labour of a hand upon the farm, for some two months now. The remains of the mamma are about half the size of the well-formed natural one—the skin is regularly contracted and adherent—the ulcers are all healed soundly, except a very small perforation, still giving exit to some discharge, of inoffensive appearance. Her general health is perfect in all respects, having recovered her former flesh, activity and cheerfulness of mind.—*Southern Med. and Surg. Journal.*

